# Acupuncture Combined with Voice Training Improved Voice Function in Patients with Primary Muscular Tension Dysphonia (MTD): A Randomized, Three-Armed, Open-Label Clinical Trial

**DOI:** 10.1155/2023/2418719

**Published:** 2023-11-03

**Authors:** Fang-Qi Liang, Yu Shi, Lei Dong, Yan Xie, Xiao-Cen Wang, Xin-Ning He, Rong Zhang, Ting Liu, Li Zhou

**Affiliations:** ^1^Hospital of Chengdu University of Traditional Chinese Medicine (Traditional Chinese Medicine Hospital of Sichuan), Chengdu 610075, China; ^2^The Second Affiliated Hospital of Army Medical University, Chongqing 400700, China; ^3^Chengdu Wenjiang District People's Hospital, Chengdu 610000, China; ^4^Chengdu University of TCM, Chengdu, China

## Abstract

**Objective:**

This randomized controlled trial aimed to evaluate the clinical efficacy of acupuncture combined with voice training for treating patients with primary muscular tension dysphonia (MTD) (Qi stagnation and blood stasis type in traditional Chinese medicine).

**Methods:**

A total of 108 patients with primary MTD (Qi stagnation and blood stasis type) were recruited in this study. The participants were randomly divided into 3 equal groups: a test group and two control groups (control groups 1 and 2). An additional 38 participants without primary MTD were recruited as the healthy group. Control group 1 received acupuncture sessions 3 times per week on alternate days on the Hegu (LI 4), Taichong (LR 3), Open Voice No. 1 point, and Open Voice No. 2 points. Control group 2 received a 40-minute voice training session once weekly. The test group received both treatments. The total treatment course for all groups was 30 days. The healthy participants did not receive any interventions. The physiological and functional voice improvements after treatment were compared between all 3 MTD groups and healthy participants. The Voice Handicap Index (VHI-10), the VHI-10 emotional score, the Chinese Medicine Symptom Score Scale (TCM), and the Grade Roughness Breathiness Asthenia Strain (GRBAS) were used to evaluate the changes in the voice after treatment. A laryngeal muscle blood oxygen monitoring was used to measure the blood oxygen saturation (SO_2_) of the suprahyoid and infrahyoid muscles, and a stroboscopic laryngoscopy was used to measure the dysphonia severity index (DSI). Acoustic voice analysis was used to measure the maximum phonation time (MPT), the jitter, and the shimmer.

**Result:**

The SO_2_ levels of the laryngeal muscle were significantly higher in the healthy subjects than in pretreatment MTD patients and were correlated with the VHI-10 and DSI scores. A significant improvement in the physiological and functional scores, the total VHI-10, the GRBAS score, the voice acoustic analysis indices, MPT, jitter, shimmer, and DSI, was noted after treatment in all 3 MTD groups (*P* < 0.05). However, the posttreatment scores in the test group were significantly higher compared to control group 2, whose score were superior than that in control group 1 (*P* < 0.05). Both the test group and control group 1 showed a significant increase in the SO_2_ levels of the laryngeal muscles after treatment, where the test group had a higher elevation than control group 1. No significant difference was noted in the posttreatment SO_2_ of the laryngeal muscles in control group 2 (*P* > 0.05).

**Conclusion:**

Acupuncture monotherapy or in combination with voice training can reduce the anxiety emotion, relieve MTD-associated systemic symptoms, and increase the SO_2_ levels of the laryngeal muscle. This combination is a promising approach for the treatment of MTD. This trial is registered with ChiCTR2200061469.

## 1. Introduction

Muscular tension dysphonia (MTD) is a hyperfunctional vocal disorder that was first identified by Morrison et al. in 1983 [1–5]. This disease is characterized by excessive tension of the external laryngeal muscles during vocalization, which alters the cartilage structure of the larynx and affects the intralaryngeal muscle tissue and vocal fold tension. MTD leads to disharmony between the vocal organs without any anatomical alternations to the vocal organs [3, 5]. A good voice is essential for effective communication. Hence, MTD markedly impairs the patient's quality of life.

The pathogenesis of MTD is still unclear, and both clinical and experimental research on MTD is scarce. Moreover, there are no specific indicators that could be used to measure the severity of MTD, and there is no effective treatment for MTD. Current treatment includes voice training, laryngeal massage, and behavioral interventions, which are mainly used to relieve symptoms and reduce the emotional anxiety caused by MTD [6–9]. Although voice training is currently recognized as an effective treatment for this disease, it has not been widely used in China.

Acupuncture is a traditional Chinese medicine (TCM) therapy that has been used to treat voice hoarseness for many years. Chinese patients prefer acupuncture rather than voice training because it is more convenient and acceptable. Acupuncture involves the insertion of small needles at specific points within the body, known as meridians. Traditional acupuncture is based on the belief that energy flows through these meridians. This energy is known as Qi. The stimulation of these meridians can reduce the Qi stagnation and blood stasis and facilitate the flow of Qi through these meridians. Acupuncture can provide a holistic, long-term improvement in the patient's general well-being by reducing the symptoms of the disease. In modern medicine, it is believed that acupuncture can stimulate the nerves underneath the skin and muscles, eventually triggering an inflammatory response that promotes tissue healing. Modern medical studies have shown that acupuncture can effectively treat hoarseness [10, 11].

Although acupuncture is a widely accepted treatment for hoarseness caused by inborn voice diseases such as chronic laryngitis, vocal fold nodules, and vocal fold polys, there is currently limited research on the effectiveness of this technique in treating hoarseness caused by MTD and other functional voice diseases. We conducted a systematic literature review including 8 studies to evaluate the efficacy of acupuncture in the treatment of primary MTD. However, only 1 of 8 studies was a clinical trial [12], and this trial had some limitations, including a small sample size, a restricted range of acupuncture interventions, and lacked outcome comparisons with a control group.

Therefore, in the present randomized controlled trial, we compared the efficacy of acupuncture alone or combined with voice therapy in the treatment of MTD.

## 2. Methods

### 2.1. Ethical Considerations

This study was approved by the Ethics Committee of the Hospital of Chengdu University of Traditional Chinese Medicine (No. 2019KL-041). Written informed consent was obtained from all patients and healthy individuals participating in this study.

### 2.2. Data Collection

Healthy individuals and MTD patients were recruited as follows: a total of 38 healthy subjects aged between 18 and 65 years with no history of hoarseness, vocal effort, pharyngeal discomfort, and laryngeal lesions were recruited. Healthy individuals that received some form of treatment for 1 month prior to the study and during the study period were excluded. Pregnant and lactating women and individuals with psychiatric disorders were excluded.

**Figure 1:**
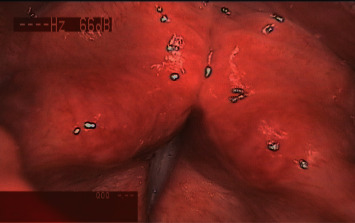
Laryngoscope view of an MTD patient with anteroposterior and lateral compression of the glottis.

MTD patients who attended the outpatient clinic of the Department of Otorhinolaryngology of the Affiliated Hospital of Chengdu University of Traditional Chinese Medicine from September 2019 to January 2021 were eligible to participate in this study. Patients aged between 18 and 65 years were included in this study if they were willing to participate in this study and met the Western medicine diagnostic criteria for primary MTD (based on clinical symptoms and laryngoscopy [13, 14]): changes in voice quality, manifesting as a tense voice, laborious pronunciation, and decreased vocal ability; persons with mild symptoms including a low voice, poor sound quality, an abnormal pitch, and difficulty pronouncing certain words; persons with severe symptoms including a harsh and rough voice, hoarseness, and aphonia; patients having the feeling of a foreign body in the pharynx, dryness, frequent throat clearing, and neck pain or tightness. Other rare symptoms may include palpitations, irritability, and anxiety. In some cases, MTD can cause overexertion of the head and neck muscles and increase the tension of the superior and inferior muscles of the neck and hyoid bone, which causes the neck muscles to bulge out. Also, it may cause distention of the jugular vein. Laryngoscopy can reveal a visible posterior fissure of the glottis, anterior-posterior or lateral compression, laryngeal ventricular vocal fold compensation in the superior glottic area, and visible glottic over closure [14]. Unlike other diseases causing hoarseness, MTD does not result in natural lesions such as hyperplasia, hypertrophy, abnormal structures, and abnormal glottic activity of the vocal fold mucosa (see Figure 1). The exclusion criteria were as follows: patients with a common cold, asthma, nervous system disorders, hearing impairment, mental illnesses, and laryngeal lesions revealed by laryngoscopy; patients treated with antibiotics, glucocorticoids, antihistamines, and other TCM promoting blood circulation and removing blood stasis; pregnant or lactating women; patients who, during the trial, violated the inclusion or exclusion criteria, used any other treatment methods, or did not comply with the treatment regime indicated by the doctor; patients with no complete clinical data; patients could not continue the treatment if they developed acupuncture sickness or other severe side effects and if they experienced a deterioration of their condition during the treatment; patients voluntarily withdrew the trial.

### 2.3. Randomization of the Participants

The random number table method was used to randomly divide the MTD patients into 3 groups: the test group, control group 1, and control group 2. According to the sample size calculation formula, the minimum sample size required for each group was 27. The baseline and demographic characteristics of the participants in the 3 MTD groups were compared to ensure the comparability.

### 2.4. Treatment Delivery

The patients in control group 1 were treated with acupuncture; those in control group 2 were treated with voice therapy; and the test group received both acupuncture and voice therapy.

### 2.5. Acupuncture Therapy

**Figure 2:**
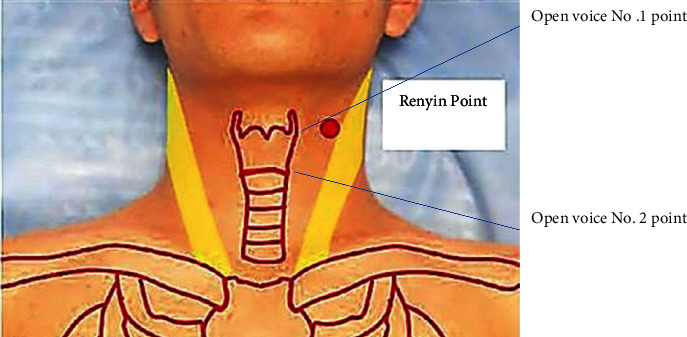
Map of acupoints (Open Voice No. 1 point and Open Voice No. 2 point).

**Figure 3:**
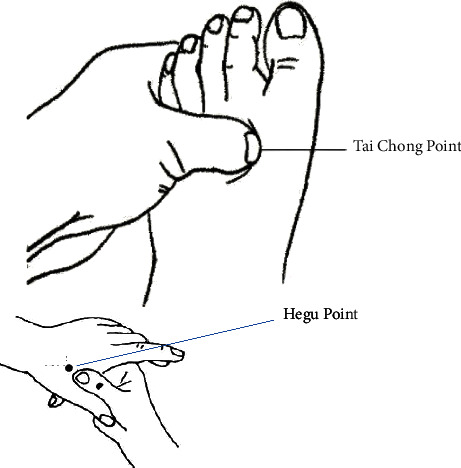
Map of acupoints (Taichong and Hegu).

The identification of treatment points and application sites was performed according to the National Standard of People's Republic of China-Meridian Point Sites (GB/T 12346-2021) [10] and *Xie's* empirical points [15] (Figures 2 and 3). The acupuncture treatment was delivered to the following 4 points:

1. Open Voice No. 1 point located at 1 inch from the upper edge of the thyroid cartilage in the midline of the neck to the outer side of the neck and at 0.5 inches from the Renying (ST 9). ST 9 was located immediately outside the upper edge of the thyroid cartilage.
2. Open Voice No. 2 point located at 1 inch from the lower edge of the thyroid cartilage in the midline of the neck and at 0.5 inches from the Shuitu (ST 10), which was located immediately outside the lower edge of the thyroid cartilage.
3. Hegu (LI 4) from *Ling Shu-Ben Lu*. It was an original point belonging to the Hand Yangming Large Intestine Meridian on the back of the hand, between the first and second metacarpal bones, at the midpoint of the radial aspect of the second metacarpal bone.
4. Taichong (LR 3) was located on the dorsal side of the foot and the posterior depression of the first metatarsal space.

All researchers were uniformly trained with the same treatment plan. The acupuncture treatment in both groups was done by qualified acupuncturist. The acupuncturist followed an aseptic procedure to disinfect their hands. The patient was then asked to lay in the supine position on the treatment bed, and the skin at the acupuncture points was disinfected with 75% alcohol and allowed to dry. Subsequently, 1-inch filiform needles (0.30 mm × 25 mm, single-use, Chengdu Honest Health Medical Equipment Co., Ltd.) were obliquely placed on the Open Voice No. 1 and Open Voice No. 2 points. The needle was twisted by 20° and slowly inserted into the space underneath the skin. If the passage of the needle was obstructed, the needle was pulled out and inserted again using a different direction. The tip of the needle was gradually inserted in the open space underneath the skin up to a maximum depth of approximately 0.7 inches. Subsequently, the needle was twisted by about 30° and kept until the patient experienced the *deqi* sensation, which was a local swelling and numbness feeling within the throat similar to that of fish bone stuck in the throat. The needle was then left in position. After that, the needle was inserted into the Hegu (LI 4) at approximately 0.5 to 0.8 inches deep until the patient felt a localized soreness and swelling spreading to the elbow, shoulder, and face. The patient was then asked to sit down on the bed, and another needle was directly inserted into the Taichong (LR 3) to a depth of approximately 0.5 to 0.8 inches until the patient felt a localized soreness and swelling radiating to the lower legs. The needles were left in the acupuncture positions for 30 minutes for each treatment session, and normal reinforcement and reduction were applied for all treatment sessions. The treatment was performed once daily on alternate days, three times a week for one month.

### 2.6. Voice Training

A trained voice rehabilitation physician performed the voice training according to the Lessac-Madsen Resonant Voice Therapy [16] guidelines. Four voice training sessions of 40 minutes each were conducted with small groups of 5 or fewer participants once a week. Participants were provided with after-class assignments and follow-up consultations via WeChat. The 4 training sessions were conducted as follows.

The first training session consisted of 4 steps. For each step, the following exercises were performed. The first step consisted of a 15-minute relaxation training whereby the patient was asked to perform chest expansion exercises, shoulder lifts, back extension, neck extension, and other exercises to relax the neck, shoulders, and back muscles. During these exercises, the patient was asked to breathe in through the nose and out via the mouth.

The second step consisted of a 5-minute throat massage with the thumbs of the 3 lateral necklines. The first line was located at the front of the neck 1 inch below the laryngeal node, and the second lateral line was located straight down the middle of the first. The third lateral line was located about half an inch below the laryngeal node and at the posterior edge of the thyroid cartilage on both sides and the thyrohyal bone.

The third exercise consisted of a 5-minute vocal fold relaxation lip trill training whereby the patient was asked to breathe in to expand the lungs and produce an airflow that makes the vocal folds vibrate. During the procedure, the patient was asked to exhale while simultaneously relaxing the lips to block the release of air and generate a series of “*dulu*, *dulu*” sounds.

The final exercise consisted of a 15-minute breathing training session divided into 2 parts. In the first part of this training session, the patients were asked to deeply inhale into the “Dantian” (a location 3 inches below the umbilicus) to protrude the abdomen and then exhale to contract the abdomen while pronouncing the “*shi*” sound for as long as possible. In the second part of this training, the patient was asked to inhale, allowing the abdomen to protrude, and then to exhale while contracting the abdomen and pronouncing the “*xu*” sound. During this process, the patient was asked to feel the power of the abdominal muscle contraction while controlling the breath through the abdominal muscle. The patient was then asked to say single words followed by short and long sentences with the palm of the hand placed 15 cm in front of the mouth to ensure that a breath was taken for every word. The patient was then asked to move on to a natural conversation.

The third training consisted of resonance training and aimed to relieve the nervous vocal mode, coordinate and enhance vocal function, and improve sound quality. This exercise was performed by resonating the *m* sound while feeling the vibration in front of the lips and a sense of relaxation in the larynx. The patient was then asked to say single words and sounds starting with the letter m, such as *mā mī mō mū māng*, *mama*, *māomī*, *mèimei*, *mùmén*, *mīmīmīmīmīmīmīmī*, and *mùmùpūpūmùmù*.

During the fourth training session, participants engaged in relaxation, breathing, and resonance exercises. These particular exercises were practiced repetitively while simultaneously reading poems and short texts and engaging in specific work-related conversations.

During the trial, no adverse events were observed following vocal training in both the test and control groups.

### 2.7. Assessment of Treatment Outcomes

The function and structure of the vocal fold were assessed before and after treatment using the following subjective and objective assessment tools.

### 2.8. Voice Handicap Index (VHI-10)

The VHI-10 is a questionnaire designed to evaluate the patient's emotional (E), physical (P), and functional (F) indicators using a rating scale ranging from 0 to 4.

### 2.9. Subjective Auditory Perception Assessment of Voice Quality (GRBAS Scale)

The GRBAS scale proposed by the Japanese Speech and Voice Society was used to classify the voice samples into four levels according to the severity of the MTD. Voice samples were scored twice independently by a listening committee composed of 3 ears, nose, and throat (ENT) specialists. The assigned GRBAS scores were compared. In cases of disagreement between the ENT specialists, the GRBAS mean score was calculated.

### 2.10. The Chinese Medicine Symptom Scale Score

This scale was self-designed according to the hoarseness disease diagnostic criteria proposed by the Otorhinolaryngology of Chinese Medicine, Classification and Identification of Articulatory Diseases, and Classification and Treatment of Articulatory Diseases by Xujiang School of Laryngology published by Jiangxi, and the Guidelines for Clinical Research on New Chinese Medicines [17]. The scale was divided into 4 primary symptoms and 2 secondary symptoms. The primary symptoms include (1) hoarseness, vocal fatigue, or pronunciation effort; (2) pain in the throat and discomfort in the pharynx when pronouncing; (3) harsh and rough sound and tension in the laryngeal muscles when trying to pronounce specific words; and (4) a dark tongue with petechial dots and a thin coating, and stringent pulse. The secondary syndromes include (1) emotional discomfort and sigh and (2) irritability. The primary symptoms were scored using a score ranging from 0 (no symptoms) to 3 (always present). The secondary symptoms were scored as either 0 (not present) or 1 (present).

### 2.11. Acoustic Parameter Analysis

The acoustic voice parameters were analyzed using Divas voice analysis software version 2.3 from Xion, Germany. For this analysis, the patients were asked to relax and breathe while pronouncing the vowels /a/, /o/, /e/, /i/, /u/, /u:/, /ä/, and /ö/. Each vowel was pronounced for over 3 seconds, with an interval between two adjacent vowels of 1 second. The middle part of the voice sample was taken. The smooth part of the voice sample was analyzed, and the jitter, shimmer, MPT, and DSI were recorded.

### 2.12. Functional Near-Infrared Spectroscopy

The INVOS 5100C local blood oxygen saturation monitor was used to simultaneously monitor the blood oxygen saturation (SO_2_) of the suprahyoid and infrahyoid muscles. For this measurement, the participants were asked to sit with their heads in a neutral position. The L1 probe was placed on the suprahyoid muscles, and the L2 probe was placed on the infrahyoid muscles. The patient was asked to read the speech test material at a normal speech rate and tone for 5 minutes. The SO_2_ values for the laryngeal muscles were recorded using the L1 and L2 probes, and the average SO_2_ value was calculated.

### 2.13. Statistical Analysis

The statistical package for social sciences software (SPSS) version 22.0 was used to analyze the data. The normally distributed variables were described by the mean ± standard deviation (±SD). The one-way analysis of variance (ANOVA) was used to compare the normally distributed continuous variables between groups. The least significant differences (LSD) *t*-test was used for the two-way comparisons between groups, and the paired *t*-test was used to compare the data within the group. The nonnormally distributed measures were described by M (P25–P75), and the Kruskal–Wallis test was used to compare variables between groups. The categorical data were described by the number of cases (percentage), and Pearson's chi-squared (*χ*^2^) test was used for the inter- and intragroup comparisons. Pearson's linear correlation analysis was used to correlate the scores between the groups. For all statistical tests, a *P* value below 0.05 was considered statistically significant.

## 3. Results

### 3.1. Baseline Data

**Figure 4:**
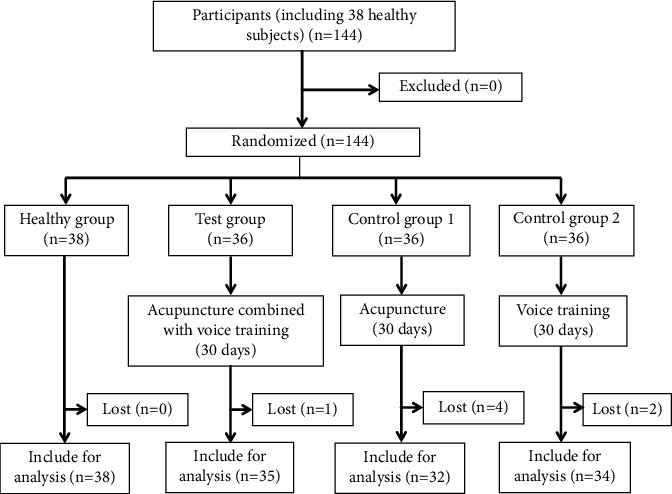
Flowchart of enrollment.

A total of 108 MTD patients and 38 healthy individuals were initially recruited for this study. The MTD patients were randomly divided into the test group, control group 1, and control group 2. However, during the trial, 1, 4, and 2 patients from the test group, control group 1 and control group 2, respectively, were lost to follow-up. A total of 139 subjects ultimately completed the study, as shown in Figure 4.

The mean ages of the MTD and healthy groups were 33.53 (±8.99) years and 34.67 (±7.84) years, respectively. The majority of the patients in the MTD (*n* = 77) and healthy (*n* = 24) groups were females. There was no significant difference in the age, gender, and other baseline data between the MTD group and the healthy group (*P* > 0.05; Table 1).

In the test group, there were 8 males and 27 females, with ages ranging from 20 to 56 years (35 ± 9 years). The disease duration ranged from 2 to 12 months (mean = 5 months). In control group 1, there were 8 males and 24 females, with ages ranging from 21 to 53 years (33 ± 9 years) with a disease duration ranging from 2.13 to 8.00 months (mean = 4 months). In control group 2, there were 12 males and 24 females with ages ranging from 21 to 52 years (33 ± 10 years) with a disease duration ranging from 2 to 8.5 months (mean = 4.5 months). The age, disease duration, and gender did not differ significantly between the 3 groups (*P* > 0.05; Table 2).

### 3.2. Correlation Analysis between Laryngeal Muscle Blood Oxygen Saturation and MTD

**Figure 5:**
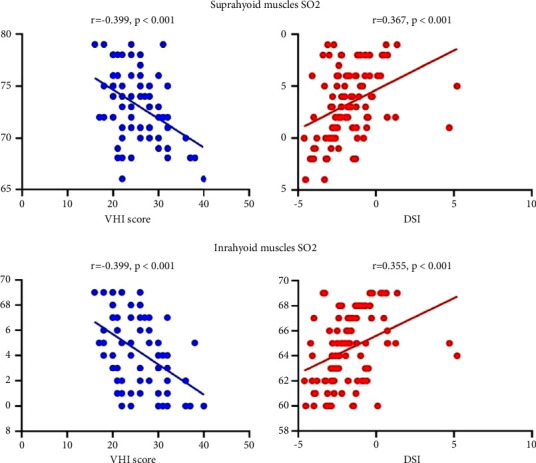
Correlations between the laryngeal muscle SO_2_ levels and the VH1 and DSI scores.

The SO_2_ of the suprahyoid and infrahyoid muscles in the pretreatment MTD groups was significantly lower than that of the normal group (*P* < 0.05) (Figure 5). A significant correlation was revealed between the SO_2_ values of the suprahyoid muscle and the VHI-10 score (*r* = 0.399) and between the laryngeal muscles SO_2_ values and the DSI (*r* = 0.367) (*P* < 0.05). Similarly, the SO_2_ value of the subglottic muscles had a significant association with the VHI-10 score (*r* = −0.422) and DSI (*r* = 0.355) (*P* < 0.05; Table 3).

After treatment, the VHI, physical, emotional, and functional scores significantly decreased from the baseline values for all 3 MTD groups (*P* < 0.05) (Table 4). The largest change for all scores was shown in the test group.

### 3.3. Comparison of the Pre- and Posttreatment GRBAS and TCM Syndrome Scale Scores between the 3 MTD Groups

After treatment, the GRBAS and TCM syndrome scale scores of patients significantly decreased relative to their pretreatment values (*P* < 0.05) for all 3 MTD groups. Notably, the test group had the largest decrease in the GRBAS scores and TCM syndrome scale scores, followed by control group 2 and control group 1 (Table 5). Moreover, the difference between the groups was statistically significant (*P* < 0.05).

### 3.4. Comparison between the Pre- and Posttreatment SO2 Levels of the Laryngeal Muscles in the Three MTD Groups

The SO_2_ values for the laryngeal muscles increased significantly after treatment for the test group and control group 1 (*P* < 0.05). However, no significant difference was detected between the pre- and posttreatment values in control group 2. The change in the SO_2_ values posttreatment did not differ significantly between the test group and control group 1 (*P* > 0.05; Table 6).

### 3.5. Comparison of the Acoustic Voice Parameters before and after Treatment among the Three MTD Groups

After treatment, a statistically significant decrease in the jitter and shimmer was noted, while the MPT and DSI significantly increased in all three groups (*P* < 0.05). These changes were more pronounced in the test group, followed by control group 1 and control group 2. The difference between the groups was statistically significant (*P* < 0.05; Table 7).

### 3.6. Safety Evaluation

No adverse events were observed in subjects in the test and control groups during the trial.

## 4. Discussion

Due to the particularity of voice disorders, voice evaluation often requires a comprehensive assessment. The assessment system can be divided into two main categories: subjective and objective, whose combination can effectively assess the severity of dysphonia, predict the nature of the disease, dynamically observe disease development, judge the treatment effect, and estimate the prognosis. Subjective assessment included the patient's evaluation of their own voice quality and quality of life (VHI-10 scale) and the physician's auditory perception assessment of the patient's voice (GRBAS). The objective assessments included the acoustic voice analysis and the laryngeal muscle blood oxygenation parameters. This study firstly adopted the objective assessments.

### 4.1. Functional Near-Infrared Spectroscopy

Our study demonstrated that the SO_2_ levels of laryngeal muscle in healthy people were significantly higher than those in MTD patients. Furthermore, the SO_2_ levels were correlated with the VHI-10 score and the DSI. These findings suggested that the laryngeal muscle oxygen parameters could be used to assess the severity of MTD and to evaluate the efficacy of MTD treatments. The NIR (near-infrared) optical local field (laryngeal muscle) blood oxygen saturation monitor used in this study provided a fast, simple, noninvasive, real-time monitoring tool to assess the SO_2_ levels of laryngeal muscles. Excessive laryngeal muscle tension is a core feature of MTD [4]. Muscle movement requires a continuous supply of oxygen to maintain aerobic energy levels. Therefore, the blood flow and laryngeal muscle SO_2_ levels may serve as good indicators of muscle function. Tension in muscles can lead to increased oxygen consumption and decreased muscle blood oxygen content. In this study, we found that acupuncture resulted in higher laryngeal SO_2_ levels posttreatment compared to voice training, indicating that acupuncture may be able to improve the metabolism of the laryngeal muscles. According to the theory of TCM, acupuncture could regulate the Qi and remove blood stasis. Blood stasis can reduce the blood oxygen content of tissues and may lead to tissue hypoxia. Therefore, acupuncture can improve tissue microcirculation and blood oxygen content.

### 4.2. Acoustic Voice Analysis; GRBAS; VHI-10

The findings of this study showed that the VHI-10 total score, physiology score, function score, GRBAS score, acoustic voice analysis indexes, MPT, jitter, shimmer, and DSI improved in all three MTD groups. However, the group treated with voice training and acupuncture achieved the best overall improvement. The effectiveness of voice training in managing MTD was confirmed in several studies [18]. Clinical research has demonstrated the effectiveness of voice training for voice disorders. Approximately 91% of otolaryngologists in the United States now recommend voice training for the treatment of nonorganic voice disorders [18]. An important concept and the first stage in voice training is relaxation training. Laryngeal massage relieves the tension of the laryngeal muscles and stimulates the secretion of the glands in the larynx, and thus reregulates the vibration patterns of the vocal fold [6]. Abdominal breathing training is another important factor in voice training which can enhance vocalization power [19, 20]. Patients can improve their vocalization through resonance training by reducing the space between the vocal folds to about 0.5 to 1 mm. The reduced space relieves the mechanical friction and collision between the vocal folds and ultimately improves the patients' voice quality.

### 4.3. VHI-10 Emotional Score

The findings of this study also showed that acupuncture resulted in a better VHI-10 emotional score and TCM syndrome scale score when compared with voice training. These findings indicated that acupuncture had obvious advantages in reducing the patient's anxiety levels and systemic MTD symptoms. According to TCM, the symptoms of MTD belong to the category of hoarseness (acoustic astringency). The disease is characterized by a sullen and monotonous voice and poor voice quality that do not relieve over time. It is often accompanied by melancholy, unhappiness, distension, pain in the dorsal ribs, emotional restlessness, sighing, and irregular menstruation [16]. In TCM, the vocal fold's meridians are believed to be linked with the liver. This theory was proposed by *Gan Zuwang*, who points out that the human vocal folds are like tendons and that the liver is the master of tendons. In addition, the liver controls the conveyance and dispersion of the whole body's Qi. The opening and closing of the vocal fold is an external manifestation of the liver regulation of Qi. Insufficient blood supply to the liver and its subsequent failure to nourish the tendon can lead to voice hoarseness. The Open Voice No. 1 and Open Voice No. 2 points were developed by the *Xujang* laryngology college. These points are located in the pharynx at the foot of the Yangming Meridian. Since these points are located closer to the larynx, they are safer and easier to access. The action of these points is closely related to the anatomical structure and physiological function of the area where they are located. The Open Voice No. 1 and Open Voice No. 2 points are widely connected to the cervical cutaneous nerve, carotid sinus, sympathetic nerve, the external branch of the superior laryngeal nerve, and the recurrent laryngeal nerve. Therefore, these acupuncture points are highly responsive and effective in promoting Qi and blood circulation and adjusting the muscle tone [21–23]. The Hegu (LI 4) and Taichong (LR 3) represent the original points of the Hand Yangming Large Intestine Meridian and the Foot Jueyin Liver Meridian. These points are mutually dependent and useful for regulating the Qi and blood, unblocking the meridians, relieving depression in the liver, and activating blood circulation to remove blood stasis.

This study carries certain limitations that warrant recognition. While the objective index employed to assess the laryngeal muscle oxygen saturation parameter demonstrated the potential connection between acupuncture treatment and the enhancement of laryngeal muscle blood oxygen content, the specific pathways through which acupuncture exerts this influence remain inadequately explored. Further investigations are therefore required to elucidate the intricate mechanisms by which acupuncture impacts laryngeal muscle blood oxygen content. Meanwhile, no follow-up observation was conducted in this study, and the durability of the voice improvement was not studied, which is expected to be further explored in future studies. In addition, due to the particularity of treatment methods, this study did not adopt blind research, but in order to ensure the accuracy of experimental data, the treatment, data collection, and data analysis evaluators of this study were independently conducted.

## 5. Conclusion

The laryngeal muscle SO_2_ levels were correlated with the severity of MTD and could be used as an objective indicator to measure the treatment efficacy. Both acupuncture and voice training can ameliorate the symptoms of MTD and the patient's anxiety, whereas their combination exhibits the optimal efficacy. Therefore, our study highlights the promising value of acupuncture in the treatment of MTD.

## Figures and Tables

**Table 1:** Baseline data comparison between the MTD group and the healthy group.

Baseline	MTD group (*n* = 101)	Healthy group (*n* = 38)	*t*/*χ*^2^	*P* value
Age (years)	33.53 ± 8.99	34.67 ± 7.84	−0.689	0.492
Sex				
Male	28 (22.9)	14 (36.8)	1.089	0.297
Female	73 (77.1)	24 (63.2)		

**Table 2:** Baseline data comparison between the three groups.

Baseline	Test group (*n* = 35)	Control group 1 (*n* = 32)	Control group 2 (*n* = 34)	*F*/*χ*^2^	*P* value
Age (years)	34.49 ± 8.58	32.75 ± 8.64	33.29 ± 9.85	0.326	0.723
Crouse (month)	5 (2–12)	4 (2.13–8)	4.5 (2–8.5)	0.496	0.780
Sex				1.505	0.471
Male	8 (22.9)	8 (25)	12 (35.3)		
Female	27 (77.1)	24 (75)	22 (64.7)		

**Table 3:** Laryngeal muscle blood oxygen saturation of participants before treatment.

Index	Group	*n*	$\bar{x}\pm \text{SD}$	*F*	*P*
Suprahyoid muscles blood oxygen saturation (%)	Test group	35	73.46 ± 3.49	209.287	<0.001
	Control group 1	32	73.00 ± 3.21		
	Control group 2	34	73.18 ± 3.39		
	Healthy group	38	90.37 ± 4.14^a,b,c^		

Infrahyoid muscles blood oxygen saturation (%)	Test group	35	64.89 ± 2.61	613.982	<0.001
	Control group 1	32	64.31 ± 2.89		
	Control group 2	34	64.29 ± 2.80		
	Healthy group	38	91.62 ± 4.47^a,b,c^		

*Note*. The SO_2_ of the superior and inferior hyoid muscles in the pretreatment MTD groups was significantly lower than the SO_2_ in the normal group (*P* < 0.05). Compared with the test group, ^a^*P* < 0.05; compared with control group 1, ^b^*P* < 0.05; compared with the normal group, ^c^*P* < 0.05.

**Table 4:** Comparison of the pre and posttreatment VHI-10 scale scores.

Group	Course	Sample	Score	Physical	Functional	Emotional
Test	Before treatment	35	25.37 ± 4.95	7.69 ± 1.51	12.89 ± 2.45	4.97 ± 1.04
	After treatment	35	8.26 ± 2.45	2.43 ± 0.81	4.23 ± 1.31	1.66 ± 0.48

Control 1	Before treatment	32	24.81 ± 4.86	7.44 ± 1.48	12.47 ± 2.46	5.00 ± 1.02
	After treatment	32	14.97 ± 2.68	4.56 ± 0.80	8.13 ± 1.64	2.69 ± 0.64

Control 2	Before treatment	34	23.74 ± 4.83	7.18 ± 1.45	12.03 ± 2.35	4.76 ± 1.02
	After treatment	34	13.56 ± 3.25	4.03 ± 0.94	6.88 ± 1.65	3.06 ± 0.81

*Note*. Compared with this group before treatment, ^*∗*^*P* < 0.05; compared with the test group, ^a^*P* < 0.05; compared with control group 1, ^b^*P* < 0.05.

**Table 5:** Comparison of GRBAS scores and TCM Syndrome Scale Scores before and after treatment in the three groups (score, $\bar{x}\pm s$).

Group	Course	Sample	GRBAS	TCM Syndrome Scale Scores
Test	Before treatment	35	11.66 ± 1.26	11.91 ± 2.17
	After treatment	35	2.80 ± 1.75	3.89 ± 1.45

Control group 1	Before treatment	32	11.53 ± 1.27	12.34 ± 2.86
	After treatment	32	6.09 ± 1.42	5.00 ± 1.30

Control group 2	Before treatment	34	11.65 ± 1.04	11.62 ± 2.41
	After treatment	34	5.21 ± 1.77	6.76 ± 1.16

*Note*. Compared with this group before treatment, ^*∗*^*P* < 0.05; compared with the test group, ^a^*P* < 0.05; compared with control group 1, ^b^*P* < 0.05.

**Table 6:** Comparison of the pre- and posttreatment blood oxygen saturation of laryngeal muscles in the three groups (%, $\bar{x}\pm s$).

Group	Course	Sample	Suprahyoid muscles	Infrahyoid muscles
Test	Before treatment	35	73.46 ± 3.49	64.89 ± 2.61
	After treatment	35	81.06 ± 3.26	72.46 ± 2.31

Control group 1	Before treatment	32	73.00 ± 3.21	64.31 ± 2.89
	After treatment	32	80.31 ± 3.24	71.22 ± 2.27

Control group 2	Before treatment	34	73.18 ± 3.39	64.29 ± 2.80
	After treatment	34	73.94 ± 3.16	65.15 ± 3.29

*Note*. Compared with the pretreatment group, ^*∗*^*P* < 0.05; compared with the test group, ^a^*P* < 0.05; compared with control group 1, ^b^*P* < 0.05.

**Table 7:** Comparison of the acoustic voice parameters before and after treatment in the 3 MTD groups (*x* ± *s*).

Group	Course	Sample	Jitter (%)	Shimmer (%)	MPT (s)	DSI
Test	Before treatment	35	1.12 ± 0.28	4.96 ± 0.601	9.55 ± 3.43	−2.08 ± 1.24
	After treatment	35	0.49 ± 0.08	3.07 ± 0.63	17.18 ± 2.87	1.89 ± 0.48

Control 1	Before treatment	32	1.06 ± 0.14	5.01 ± 0.61	9.33 ± 2.98	−1.74 ± 1.39
	After treatment	32	0.65 ± 0.12^a^	3.67 ± 0.46^a^	11.60 ± 2.30^a^	1.07 ± 0.65^a^

Control 2	Before treatment	34	1.04 ± 0.19	5.01 ± 0.69	9.90 ± 3.58	−1.86 ± 1.34
	After treatment	34	0.56 ± 0.12^a,b^	3.36 ± 0.54^a,b^	15.45 ± 2.22^a,b^	1.41 ± 0.70^a,b^

*Note*. Compared with this group before treatment, ^*∗*^*P* < 0.05; compared with the test group, ^a^*P* < 0.05; and compared with control group 1, ^b^*P* < 0.05.

## Data Availability

The original data used to support the findings of this study are available from the corresponding author upon request.
